# Efficacy of inpatient pulmonary rehabilitation in elderly patients with acute exacerbation of COPD: randomised controlled trial study

**DOI:** 10.1007/s11845-026-04343-w

**Published:** 2026-04-15

**Authors:** Marwa Mohammed, Taha Hussein Musa, Temitope Emmanuel Komolafe, Fatma Gamal Elsayed Ibrahim, Hanaa Sayed Abdellateef Gahamy, Sanaa Fathy Kotb, Hala Sabry Alsaedy, Tamer I. Abo Elyazed

**Affiliations:** 1 Faculty of Physical Therapy- Beni-Suef University, 62521 Beni-Suef, Egypt; 2 Pharmacy Program,School of Medical and Health Science , Libyan International Medical University, Benghazi, Libya; 3 Collaborative Research Centre, Shanghai University of Medicine & Health Sciences, 201318 Shanghai, China; 4 Faculty of Medicine for Girls- Al-Azhar University, Cairo, 11754 Egypt; 5 Faculty of Physical Therapy - October 6 University, 12585 Giza, Egypt; 6 School of Medicine, Darfur University College, Nyala, Sudan

**Keywords:** Inpatient pulmonary rehabilitation, Patient-Centered Outcomes, Fatigue, Sleep Quality

## Abstract

**Background:**

Chronic Obstructive Pulmonary Disease (COPD) is a leading cause of morbidity and mortality worldwide. Data on Acute exacerbation of COPD (AECOPD) among older adults is scarce. The study aims to assess the impact of early initiation of pulmonary rehabilitation (PR) during hospitalization on exercise capacity, dyspnea, sleep quality, fatigue, and the length of hospital stay in patients with AECOPD.

**Methods:**

A single-blind randomised controlled trial. The study took place in the Chest Diseases Department at Alzahraa University Hospital, Cairo, Egypt, between June and September 2025.

Subject: 60 patients completed the study, randomised into two equal groups. Group of pulmonary rehabilitation (PR) and control group each included 30patients with a ratio of 1:1.

**Results:**

There were notable significant differences between groups; the PR group showed more improvement in walking distance measured by the 6-minute walk distance (6MWD), fatigue, sleep quality, dyspnea, and patients spent less time in the hospital. Our analysis found that fatigue and dyspnea were closely linked and central to patients’ experiences. Dyspnea was a stronger predictor of functional capacity than forced expiratory volume at 1st second (FEV1).

**Conclusion:**

Overall, these findings demonstrate that inpatient PR is effective in improving outcomes and physical performance while reducing healthcare utilization in elderly patients with AECOPD.

## Introduction

Chronic Obstructive Pulmonary Disease (COPD) is a public health concern and a major cause of mortality globally, with nearly 90% of related deaths in low- and middle-income countries (LMICs) [1, 2]. Earlier studies show that older individuals with COPD are especially susceptible to acute exacerbations (AECOPD), which are often linked to progressive decline in pulmonary function, higher risk of respiratory failure, and increased mortality rates [3].

AECOPD can quickly undermine a patient’s health. These episodes often result in hospital stays. Hospitalisation outcome reported lung decline, an increase in muscle mass loss, a decrease in the quality of life (QOL), and an increased risk of death among the patients [4]. The increasing rate of population ageing was followed by an increasing number of elderly people who are reported to have issues of AECOPD. Healthcare systems are overstretched, and treatment costs continue to rise. For this reason, there is a need to meet these global challenges through national and international organisations. Moreover, international respiratory societies are urgently in search of innovative and cost-effective ways out. It is fundamental for healthcare workers, health professionals, and policymakers to collaborate towards developing effective instrument strategies for addressing the described challenges [4].

More evidence from published literature shows that the AECOPD reports quickly degenerate symptoms and often require additional treatment [5]. Thus, the use of pharmacological and non-pharmacological interventions, in addition to pulmonary rehabilitation (PR), was considered a primary source of option [6]. Reported evidence highlights that PR alleviates symptoms in COPD patients [7]. Early initiation of inpatient PR has proven its safety and feasibility in elderly patients with AECOPD [8]. Remarkably, the outcomes of a two-week inpatient rehabilitation program have been shown to improve knee extensor muscle strength in elderly individuals recovering from AECOPD [9]. In addition, the use of exercise training, a central component of PR, is widely recognised not only as the foundation of PR but also as an accessible strategy for disease symptom management and prevention, cost-effectively [9].

While most existing research has focused on outpatient rehabilitation in the elderly following AECOPD, studies investigating inpatient PR remain limited [10, 11] Further studies on AECOPD have verified that a short-term inpatient rehabilitation protocol, administered twice daily for 10 to 30 min, over four consecutive days, in a group of elderly patients, leads to significant improvements in dyspnea, exercise tolerance, cough, and sputum expectoration [10]. An additional study was conducted to compare a two-week inpatient program in elderly patients with AECOPD that includes blood flow restriction resistance exercise (BFR-RE) with a similar programme without BFR. The outcomes showed better muscle strength and balance, even though no significant changes were reported in exercise tolerance [11]. Despite these promising outcomes, the specific effects of early inpatient rehabilitation in elderly patients with AECOPD remain insufficiently explored among elderly people with AECOPD. The present study aims to assess the impact of early inpatient PR on exercise tolerance, dyspnea, sleep quality, fatigue, and hospital discharge timing in this vulnerable population.

## Methods

### Participants

A prospective, single-blind, randomised controlled trial (RCT) was conducted at Alzahraa University Hospital, within the inpatient Chest Diseases Department in Cairo, Egypt. The whole study duration was conducted from June to September 2025. Participants were randomly assigned to either the rehabilitation or control group in a 1:1 ratio using computer-generated allocation sequences. Group codes were organized in serially numbered blocks and concealed in sealed, opaque envelopes stored in a secure office. Healthcare providers responsible for patient care were blinded to group assignments to minimize bias. A total of 102 patients diagnosed with mild-to-moderate COPD classified as GOLD stage II–III, with a forced expiratory volume in one second (FEV₁) ranging from 50% to more than 80% of predicted values, were enrolled following spirometric confirmation by specialized chest physician.

### Sample size calculation

Before enrollment, we calculated the required sample size with Epi Info (version 7.2). Based on previous studies, we selected exercise tolerance, measured by the six-minute walk distance (6MWD), as the primary outcome, using a mean difference of 45.27 m. This analysis indicated that a minimum of 30 contributors per group was needed to achieve a 95% confidence interval and 90% statistical power, accounting for a 20% dropout rate [12] .

## Inclusion criteria

Participants were eligible for enrollment if they met the following conditions: [1] diagnosis of mild to moderate AECOPD [2], age ≥ 65 years [3], clear level of consciousness, and [4] presence of dyspnea not attributable to cardiac conditions, pneumothorax, or pulmonary oedema.

## Exclusion criteria

**Fig. 1:**
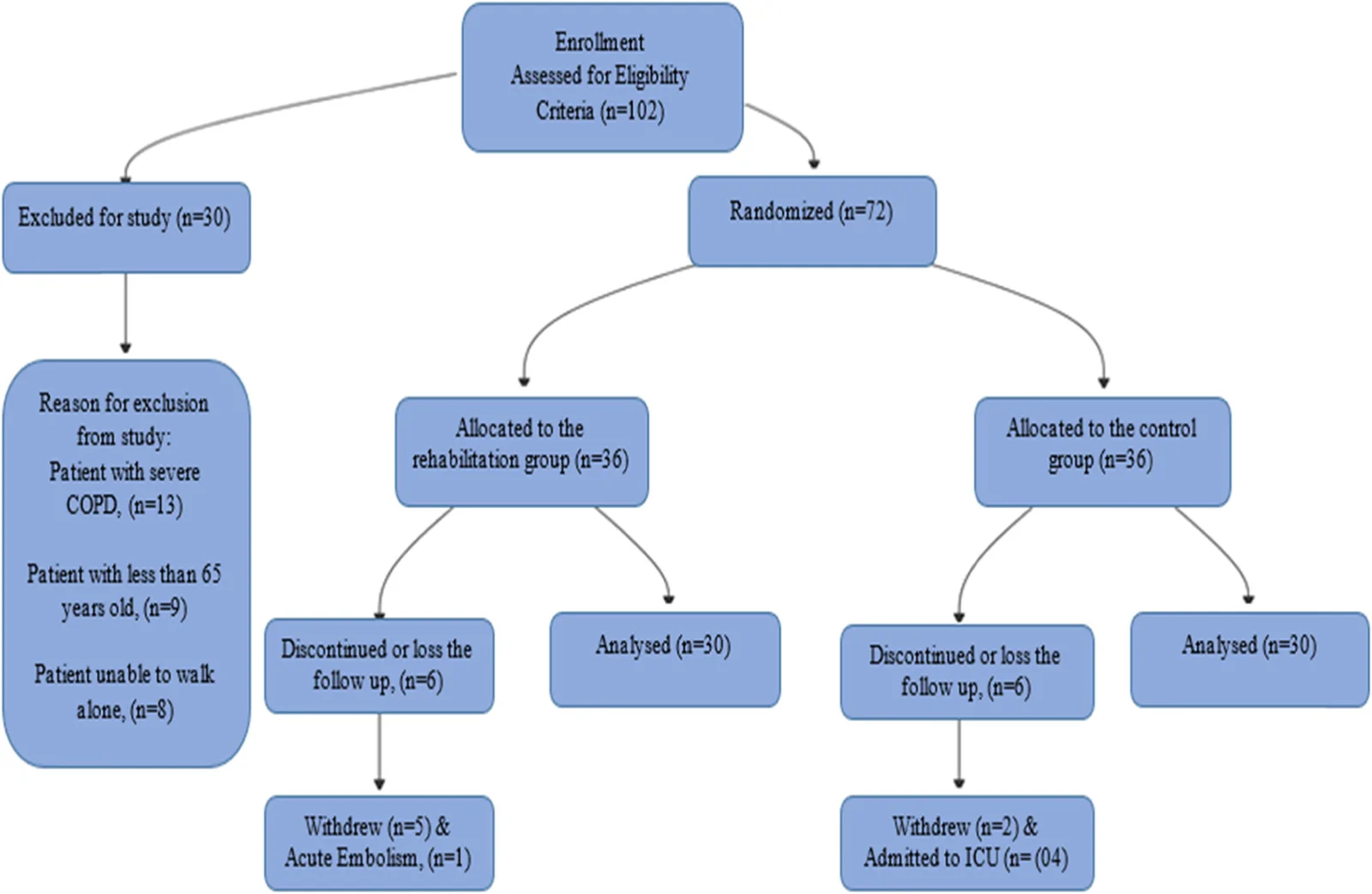
Flowchart of participant selection after application of inclusion/exclusion criteria

Patients were excluded from the study owing to the following conditions: [1] he or she reported systolic blood pressure < 90 mmHg [2], unstable psychological status [3], hemoptysis [4], pneumothorax [5], pulmonary oedema, or [6] dependence on mechanical ventilation. A total of 102 patients with COPD were assessed for eligibility. Of these numbers, 30 participants were excluded, and the remaining 72 who met the inclusion criteria were randomised into two groups: the rehabilitation group (*n* = 36) and the control group (*n* = 36). Twelve participants discontinued the study. The inclusion and exclusion criteria are shown in Fig. 1.

## Ethical considerations

The study received Ethical approval from the Ethics Committee of the Faculty of Medicine for Girls at Al-Azhar University, Cairo, Egypt, and was granted ethical permission for the study. Approval number (FMG-IRB 2025032724). The lead investigator thoroughly explained the study’s objectives, methods, and eligibility requirements to participants before recruitment. Each participant was informed that they might withdraw from the trial at any time if they felt unwell. Before recruitment, all subjects provided informed consent, and all study methods were in accordance with the Declaration of Helsinki’s requirements. The trial was registered with the ClinicalTrials.gov number NCT07020182.

## Clinical measurements

### Spirometry

The diagnosis of COPD was confirmed by spirometry, demonstrating evidence of non-fully reversible airflow obstruction, which was defined as a post-bronchodilator forced expiratory volume at 1st second /Forced Vital capacity(FEV₁/FVC) ratio of less than 0.70 [13]. Patients were included if they had been hospitalized for more than 24 h with a diagnosis of COPD in accordance with the Global Initiative for Obstructive Lung Disease (GOLD) guidelines. Medical management was directed following the GOLD 2025 references. The diagnosis of AECOPD was based on clinical presentation and exacerbation severity. Following a comprehensive assessment, including the patient’s medical history, physical examination, pulse oximetry, sputum culture, and chest radiography, conducted by a chest physician [14]. Spirometry was performed using a mass flow spirometer (Smart PFT Co Transfer, made in Germany). Spirometric parameters, including FEV₁, FVC, and FEV₁/FVC ratio, were calculated in accordance with the American Thoracic Society (ATS) guidelines [10]. The outcome of measurements was reported both as absolute values and as percentages of predicted norms.

### The 6-Minute Walk Test (6MWT)

The 6MWT is a widely utilized tool to assess functional status in patients and serves as a reliable predictor of morbidity and mortality in individuals with COPD [15]. Its reproducibility in this population has been established, with an intra-class correlation coefficient of 50.93 [16]. The test was conducted in accordance with the American Thoracic Society (ATS) guidelines, using a 30-meter corridor with standardized verbal encouragement throughout the procedure [17]. All participants received instructions in standard Arabic and were asked to walk as far as possible within six minutes. Each patient completed the test twice on the same day, with a one-hour rest interval between trials. The maximum distance achieved in either test was recorded for analysis.

### Fatigue Severity Scale (FSS)

Fatigue parameters were assessed using a self-administered questionnaire comprising nine items, each rated scale from 1 to 7. Lower total scores reflect reduced fatigue levels, whereas higher scores indicate greater fatigue severity [18]. The overall score was calculated by dividing the total by nine; a mean score exceeding 4 points was considered indicative of clinically significant fatigue [19].

### Modified British Medical Research Council (mMRC)

The mMRC dyspnea scale is widely recognized as a simple, valid, and reliable tool for evaluating breathlessness in patients with COPD [20]. A score of ≥ 2 on the mMRC scale is commonly used to distinguish between lower and higher levels of dyspnea severity. For severe physical inactivity, the sensitivity and specificity increase to 81% and 66%, respectively, while sedentary behavior is associated with 61% sensitivity and 70% specificity [20].

### Pittsburgh Sleep Quality Index (PSQI)

PSQI is a self-administered instrument designed to evaluate sleep quality over the preceding month. It comprises 19 items that yield seven component scores: subjective sleep quality, sleep latency, sleep duration, habitual sleep efficiency, sleep disturbances, use of sleep medication, and daytime dysfunction. These components are summed to produce a global score, with values greater than 5 indicating poor sleep quality [21].

### Study outcomes

The primary outcome of the study was to evaluate the effect of PR, compared to standard care without intervention, on exercise capacity. Secondary outcomes included assessing the comparative efficacy of PR versus the control group in relation to dyspnea severity, sleep quality, fatigue levels, and duration of hospital stay.

### Exercise program

The experimental group received an inpatient PR program. This included resisted breathing exercises using a threshold inspiratory muscle trainer, low-load resistance training at 15%–30% of one repetition maximum (1-RM) as tolerated and supervised aerobic exercise in the form of walking, daily sessions 7 days /week. The aerobic sessions were guided by a physiotherapist and targeted a rate of perceived dyspnea (RPD) between 4 and 6 on a 10-point scale, with a duration of 20 min or adjusted based on individual tolerance [11]. In contrast, the control group received standard medical treatment prescribed by their physician without any rehabilitation intervention.

### Statistical analysis

The IBM^®^ **SPSS** version 25.0 for Windows was used for statistical analysis. Mean and Standard Deviation (mean ± SD) were reported for normally distributed variables or continuous variables. The difference Chi-square test between categorical variables and the independent t-test for continuous variables. The median and interquartile range were reported for patients by Hospital Length of Stay. Pearson’s correlation coefficients were used to assess the relationship between changes in key continuous variables (walk distance, fatigue, dyspnea, and sleep quality) were assessed using Pearson’s correlation. A multiple linear regression model was applied to determine predictors of improvement in walk capacity. Statistical significance for all two-tailed tests was established by a p-value < 0.05. All statistical tests were two-tailed, and statistical significance was set at *p* < 0.05.

### Baseline demographic and clinical characteristics

The analysis of baseline demographics and clinical features showed that the randomisation variables. The calculation revealed several statistically significant differences reported. No differences between groups were observed in age, weight, BMI, and distribution of categories of BMI (*P* > 0.05). The prevalence of major comorbidities, hypertension and diabetes was also equal. Critically, objective measures of disease severity like lung function values (FEV_1_, FVC, FEV_1_/FVC ratio) and resting oxygen saturation were also not different at baseline. Systolic blood pressure was also comparable. However, significant differences were present in other areas. The control group contained a substantially higher proportion of housewives and never-smokers, while the intervention group included more employers and former smokers. Most importantly, there was a staggering difference in baseline symptoms: the intervention group demonstrated significantly worse fatigue and more dyspnea before the intervention began. In addition, the control group had a statistically lower diastolic blood pressure and larger resting pulse rate at baseline (Table 1).

**Table 1:** Baseline demographic and clinical characteristics of the study participants

Characteristic	Group A (Rehabilitation) (*n* = 30)	Group B (control) (*n* = 30)	*p*-value
Gender n (%)			
Male	14 (23.3)	10 (16.7)	0.292
Females	16 (26.7)	20 (33.3%)	
Age, years	69.6 ± 3.1	68.8 ± 4.5	0.461
Weight, kg	79.0 ± 8.8	76.5 ± 6.5	0.208
BMI, kg/m²	28.4 ± 3.4	29.4 ± 4.7	0.345
Education Level, n (%)			0.148
Not educated	12 (40.0)	18 (60.0)	
Primary school	14 (46.7)	12 (40.0)	
Middle school	2 (6.7)	0 (0.0)	
High school	2 (6.7)	0 (0.0)	
Occupation, n (%)			0.004*
Housewife	16 (53.3)	24 (80.0)	
Employer	10 (33.3)	0 (0.0)	
Worker	4 (13.3)	4 (13.3)	
Driver	0 (0.0)	2 (6.7)	
Comorbidities, n (%)			
Hypertension	22 (73.3)	20 (66.7)	0.573
Diabetes	8 (26.7)	10 (33.3)	0.573
Smoking Status, n (%)			0.066
Never Smoker	16 (53.3)	24 (80.0)	
Former Smoker	10 (33.3)	2 (6.7)	
Current Smoker	4 (13.3)	4 (13.3)	
BMI Category, n (%)			0.959
Normal body weight	6 (20.0)	6 (20.0)	
Overweigh	13 (43.3)	12 (40.0)	
Obese	11 (36.7)	12 (40.0)	
Lung function parameters			
FEV_1_, L	1.03 ± 0.68	1.03 ± 0.32	0.961
FVC, L	1.89 ± 1.03	2.01 ± 0.46	0.562
FEV_1_/FVC %	52.5 ± 7.0	50.6 ± 7.7	0.329
Baseline symptoms			
Fatigue severity scale pre	51.7 ± 2.16	32.73 ± 5.82	< 0.001*
mMRC pre	3.43 ± 0.49	1.93 ± 0.61	< 0.001*
6MWT pre	179 ± 54.7	108 ± 20.6	0.348
Hospital stays per days	13.2 ± 3.92	16.6 ± 3.98	0.419
Vital Signs			
Oxygen Saturation (SpO₂), %	94.4 ± 1.5	94.3 ± 1.0	0.685
Systolic BP, mmHg	132.0 ± 11.9	128.0 ± 8.5	0.138
Diastolic BP, mmHg	86.3 ± 8.8	80.7 ± 8.1	0.012*
Pulse Rate, bpm	86.9 ± 6.3	91.4 ± 3.1	< 0.001*

**Statistically significant difference (*p* < 0.05) *; BMI: Body mass index. FVC: Forced Vital Capacity.FEV1: Forced expiratory volume at 1st second. mMRC: Modified Medical Research Council

### Changes in patient-centered outcomes following pulmonary rehabilitation

The pulmonary rehabilitation intervention produced highly significant improvements across all outcomes. Participants experienced a dramatic reduction in symptom burden, with fatigue scores decreasing by a clinically important 19.0 points and dyspnea severity (mMRC) being cut by half (1.5 points) at 95% CI [-0.53 (-0.81 to -0.26)], representing another large and clinically significant improvement. Functional capacity showed a major improvement, with the 6MWD increasing by 42.3 m at 95% CI [14.40 (2.69 to 26.11)], exceeding the established threshold for clinical significance in COPD patients, confirming a large and clinically significant improvement in functional exercise tolerance. A statistically significant improvement in sleep quality was also observed. These results demonstrate the intervention’s profound efficacy in improving both physical performance and key patient-reported symptoms (Table 2).

**Table 2:** Changes in patient-centered outcomes following pulmonary rehabilitation

Outcome Measure	Pre-Test Mean (SD)	Post-Test Mean (SD)	Mean Change (95% CI)	*p*-value	Clinical Interpretation
Fatigue Score	51.7 (2.2)	32.7 (5.8)	19.0 (17.6 to 20.3)	< 0.001	Large Clinically Significant Improvement.
6MWT Distance (m)	143.3 (54.3)	185.6 (56.9)	42.3 (36.2 to 48.4)	< 0.001	Large Clinically Significant Improvement
PSQI (Sleep Quality)	12.6 (2.7)	10.2 (3.4)	2.4 (1.9 to 2.8)	< 0.001	Statistically Significant Improvement
mMRC Dyspnea	3.4 (0.5)	1.9 (0.6)	1.5 (2.4 to 2.7)	< 0.001	Large Clinically Significant Improvement.

### Comparison of clinical outcomes following PR versus standard care

The results indicated that PR intervention yielded superior patient outcomes compared to standard care. The PR intervention demonstrated a significantly greater improvement in the primary endpoint of exercise capacity, with a mean difference of 14.40 m (95% CI: 2.69–26.11, *p* = 0.017) in 6MWT. This pattern was consistently observed across all secondary outcomes, with the PR intervention showing substantially greater reduction in fatigue (-9.27 points, *p* < 0.001), improved sleep quality (-2.27 points, *p* < 0.001), and enhanced dyspnea scores (-0.53 points, *p* < 0.001). These consistent findings demonstrate that PR intervention was associated with better functional and symptomatic improvements than the standard care program in this patient population (Table 3).

**Table 3:** Comparison of clinical outcomes following PR versus standard care

Variable	Group A (Rehabilitation) (*n* = 30)	Control Group (*n* = 30)	Mean Difference (95% CI)	t-value	*p*-value
6MWD	49.47 ± 23.26	35.07 ± 22.02	14.40 (2.69 to 26.11)	2.46	0.017
Fatigue	-23.60 ± 2.21	-14.33 ± 2.78	-9.27 (-10.56 to -7.97)	-4.28	< 0.001
PSQI	-3.50 ± 1.38	-1.23 ± 1.55	-2.27 (-3.03 to -1.51)	-5.98	< 0.001
mMRC	-2.77 ± 0.50	-2.23 ± 0.57	-0.53 (-0.81 to -0.26)	-3.84	< 0.001

### Multiple regression analysis

This study identified gender and change in dyspnea as the only independent predictors of baseline 6MWT distance. Men walked a longer distance than women are in accordance with normative data and physiological expectation, as men tend to have more muscle mass and aerobic capacity. The most striking clinical observation is the strong relationship between dyspnea and functional capacity. Change in dyspnea (mMRC_change) and baseline value (mMRC pre) were both significant determinants of walking distance. This emphasizes that the subjective feeling of breathlessness in a patient is a very significant limiting factor in exercise performance and, in many instances, more significant than some objective physiological parameters. The lack of significance of FEV_1_ and SpO₂ is interesting. It suggests that in this patient population, functional performance is more closely linked to symptomatic burden (dyspnea) than to these traditional measures of lung function and oxygenation (Table 4).

**Table 4:** Multiple regression analysis results. Predictors of baseline using the standardized 6MWT

Predictor Variable	B (Coefficient)	Std. Error	t-value	*p*-value
Male	19.978	15.384	-2.276	0.027*
mMRC_change	-29.153	12.809	-2.081	0.043*
mMRC pre	-36.011	17.301	-1.956	0.056
Age	2.079	1.794	0.586	0.560
FEV_1_	9.007	16.881	1.158	0.252
SPo2	0.925	6.086	0.534	0.596
Fatique scale pre	-7.389	3.777	-1.956	0.880
BMI (kg/m^2^)	-8.555	9.255	-0.924	0.200
FEV_1_/FVC_1_	0.906	1.230	0.737	0.360

## Discussion

The findings demonstrated statistically significant improvements in exercise tolerance (as measured by the 6MWT, fatigue levels, dyspnea severity, and sleep quality in the experimental group compared to the control group. Additionally, the intervention group experienced a shorter hospital stay, with no reported adverse events. All outcomes reached significance at *p* < 0.05.

The findings of our study are consistent with those of a recent systematic review, which reported that inpatient PR significantly enhances 6MWT performance, five-repetition sit-to-stand test outcomes, and overall QOL, primarily through improvements in lower-limb muscle strength, without affecting hospital length of stay [22].The more pronounced improvements observed in our study may be attributed to the relatively younger age and milder disease severity of our participants compared to those in the referenced review (mean age = 76 ± 10 years; mean FEV₁% = 49% ± 24%) [22]. In contrast, other studies have yielded differing results. For instance, a two-week inpatient rehabilitation program incorporating BFR-RE showed no significant change in 6MWT when compared to a matched resistance exercise protocol without BFR in post-AECOPD patients [11]. Although that study demonstrated statistically significant gains in muscle strength and Short Physical Performance Battery (SPPB) scores, walking distance remained unchanged [11] .

The superior outcomes in our study may be partially explained by the inclusion of inspiratory muscle training (IMT) within the rehabilitation protocol. IMT allowed for objective monitoring of training loads and employed a non-specific approach that facilitated recruitment of the diaphragm and accessory inspiratory muscles in response to external mechanical loading [23]. Previous studies have shown that IMT can improve functional capacity, with reported gains in 6MWT of 66 m and 48 m, respectively [12, 24]. The integration of IMT with BFR-RE may offer synergistic benefits, enhancing both muscle strength and walking capacity in elderly inpatients recovering from AECOPD. However, further research is warranted to substantiate these findings.

In terms of exercise tolerance assessed by 6MWT, both groups demonstrated significant improvements in walking distance, with the intervention group showing a greater mean increase of 14.40 m (95% CI: 2.69–26.11, *p* = 0.017). Notably, the improvements in both groups exceeded the MCID of 30 m [25], suggesting that initiating inpatient PR during hospitalization may enhance exercise capacity in patients with AECOPD. The observed gains in the control group may be partially explained by baseline demographic differences, including a higher proportion of housewives and never-smokers, compared to the intervention group, which comprised more employed individuals and former smokers. Additionally, the control group presented with lower baseline fatigue severity scores and lower mMRC dyspnea scores (32.73 ± 5.82 and 1.93 ± 0.61, respectively), which may have contributed to their favourable response.

The study revealed a significant reduction in fatigue severity among participants in the rehabilitation group (− 23.60 ± 2.21) compared to the control group, with a 95% CI of − 9.27 [− 10.56 to − 7.97] and a *p*-value < 0.001. Muscle fatigue is defined as a reversible decline in maximal voluntary contraction (MVC) force during sustained activity and can be further categorized into central fatigue, characterized by reduced voluntary muscle activation (VA) and peripheral contractile muscle fatigue (pMF) [26]. These findings align with previous research demonstrating that exercise training enhances walking capacity and mitigates peripheral muscle fatigue, indicating a clear trend toward reduced fatigability following rehabilitation [26].

Improved fatigue tolerance likely contributed to enhanced exercise performance, as prior studies have shown that hospitalized patients with AECOPD tend to remain sedentary during admission and exhibit persistently low activity levels up to one-month post-discharge. Reduced physical activity is a recognized risk factor for hospital readmission in this population [27, 28]. Moreover, hospitalization is associated with rapid declines in muscle strength, with quadriceps cross-sectional area decreasing by up to 5% within five days. Evidence supports the efficacy of inpatient PR in preserving lower limb muscle strength [22]. For instance, one study reported a 10% increase in quadriceps force after seven days of daily resistance training using a knee extension chair, compared to a 1% gain in the control group [29]. Another investigation involving elderly patients demonstrated an 8% improvement in muscle strength following a two-week inpatient BFR-RE program, versus 5.6% in the control group (*p* = 0.3) [11]. Enhancing exercise tolerance during hospitalization or shortly after discharge remains a critical therapeutic goal in the management of AECOPD, and pulmonary rehabilitation plays a pivotal role in achieving this outcome.

A significant reduction in dyspnea was observed in both groups, as measured by the mMRC scale, aligning with findings from previous studies [30] .Notably, our results contrast previous study found that patients with mMRC grade 5 exhibited less improvement compared to those with lower baseline dyspnea scores [31]. In our cohort, dyspnea scores improved markedly from 4.4 ± 0.5 to 1.9 ± 0.06, representing a greater change than previously documented [30]. This enhanced response may be attributed to several factors: participants in our study had moderate COPD severity, received daily inpatient rehabilitation during a relatively short hospital stay, and despite were older than those in the referenced study. Furthermore, the extended duration of our intervention may have contributed to the improvement observed. It is well established that patients presenting with higher baseline dyspnea, poorer functional capacity, and lower health status tend to respond more favorably to PR [7].

Our findings are consistent with previous research indicating that inpatient PR can positively influence length of hospital stay (LOS) [32]. However, other studies have reported that while inpatient PR may not significantly reduce LOS, it does not appear to prolong hospitalization in patients with AECOPD [22, 33, 34]. An earlier study documented LOS ranging from 2 to 25 days, with a mean duration of 6.69 ± 4.02 days. Key factors associated with extended LOS included the number of comorbidities (*p* = 0.007), elevated blood eosinophil counts at admission (*p* = 0.022), and the need for mechanical ventilatory support (*p* < 0.001) [35]. Prolonged hospitalization has been linked to increased frailty and greater post-discharge healthcare needs [22].

Skeletal muscle dysfunction is a recognized predictor of mortality in COPD, yet few clinical trials have examined muscle function as a primary outcome following PR during AECOPD. Reduced muscle strength has been associated with higher mortality rates in patients with moderate to severe COPD [36]. Despite growing interest, there remains a notable gap in the literature regarding the impact of inpatient PR on LOS and muscle function outcomes in this population.

The improvement in sleep quality observed following inpatient rehabilitation in our study aligns with previous findings indicating that exercise interventions can enhance sleep outcomes. Poor sleep quality in individuals with COPD has been linked to greater disease severity, including elevated dyspnea levels and reduced QOL [37]. Cross-sectional analyses have further associated sleep disturbances with symptoms such as cough, dyspnea, and higher COPD Severity Scores [38]. Sleep impairment is prevalent among COPD patients and is frequently correlated with advanced disease stages and deteriorated health status, contributing to a negative impact on overall QOL [39].Although no prior studies have specifically examined the effect of PR on sleep quality in COPD populations, the improvements noted in our cohort may be indirectly attributed to enhanced dyspnea control and increased exercise capacity, as reflected in 6MWT performance. However, QOL metrics and post-rehabilitation lung function parameters were not assessed in this study, limiting our ability to determine whether changes in COPD severity contributed to sleep improvements. These findings underscore the importance of addressing sleep quality as a critical component in future COPD rehabilitation research.

Our findings identified several predictors of baseline 6MWT, including male gender, changes in mMRC score, baseline mMRC score, age, FEV₁, oxygen saturation (SpO₂), fatigue level, BMI, and FEV₁/FVC ratio. These variables were significantly associated with initial exercise capacity. In contrast, previous studies have reported that predictors of 6MWT response to PR include age, pre-rehabilitation 6MWT distance, end-of-test dyspnea before PR, and the use of long-term oxygen therapy. Specifically, older age, greater pre-PR walking distance, higher dyspnea scores at the end of the pre-PR test, and oxygen supplementation were linked to reduced improvements in post-PR 6MWT performance [40].Additional research has highlighted physiological characteristics that may predict enhanced physical performance following PR in COPD patients. Individuals with higher body weight, increased body fat (without obesity), greater dry lean mass, elevated haemoglobin levels, higher erythrocyte counts, increased hematocrit, and elevated iron levels were shown to derive greater benefit from rehabilitation interventions [41] .

Although body composition was not assessed in either group following PR in our study, future research is warranted to explore its impact, particularly given the reported prevalence of sarcopenia among patients with COPD, which stands at 21.6% (95% CI: 14.6–30.9%, I² = 94%). Rates vary from 8% in population-based studies to 21% in clinic-based cohorts, and up to 63% among COPD patients residing in nursing homes [42]. While numerous predictors of PR success have been identified, their applicability and comparability across studies remain limited due to variations in program design, patient characteristics, duration, setting (inpatient vs. outpatient), and outcome measures [41].

An earlier study illustrated that ventilatory reserve, inspiratory strength, and peripheral muscle strength emerged as significant predictors of training efficacy [29]. Furthermore, another study employed multidimensional response profiling to identify a “very good responder” cluster. Patients in this group exhibited more pronounced dyspnea symptoms, a higher frequency of hospitalizations within the previous year, poorer physical performance, lower satisfaction with daily activities, increased symptoms of anxiety and depression, and overall poorer health status. Notably, this cluster also included a greater proportion of individuals who had undergone inpatient PR compared to other response groups [7]. These findings reinforce the therapeutic value of PR programs for patients recovering from COPD exacerbations.

### Strengths of the study

This study demonstrates that in-hospital exercise rehabilitation for patients admitted with AECOPD is both safe and effective, with no reported adverse events or mortality. The intervention featured individualised exercise prescriptions tailored to each patient’s needs and was delivered under the direct supervision of qualified physiotherapists. These findings support the integration of PR into acute care protocols during hospitalization for AECOPD.

### Limitations of the study

The study was conducted at a single centre and involved elderly patients with mild to moderate AECOPD, which may limit the generalizability of the findings to broader COPD populations. Future research should investigate the efficacy of exercise-based rehabilitation programs initiated during hospitalisation and continued into the post-discharge period, with outcome assessments conducted at discharge and follow-up to distinguish between in-hospital and post-hospital effects. Additionally, studies involving diverse age groups and varying degrees of AECOPD severity are recommended to enhance external validity.

## Conclusion

The pulmonary rehabilitation intervention yielded substantial clinical benefits, including marked improvements in fatigue, dyspnea, and functional capacity, as well as statistically significant enhancements in sleep quality and a reduction in hospital length of stay. These findings underscore the effectiveness of inpatient rehabilitation in alleviating core symptoms and promoting physical recovery in patients with AECOPD. Furthermore, the analysis revealed that male gender and lower baseline dyspnea severity, particularly improvements in dyspnea symptoms, were predictive of greater functional walking capacity as measured by 6MWT. Clinically, this highlights the critical importance of targeting dyspnea relief to optimise mobility and functional outcomes in COPD management.

## Abbreviations

COPD: Chronic Obstructive Pulmonary Disease
PR: Pulmonary rehabilitation
6MWT: The six-minute walk test
mMRC: Modified British Medical Research Council
QOL: Quality of life
BFR-RE: Blood flow restriction resistance exercise
FEV₁: Forced expiratory volume in 1^st^ second
FSS: Fatigue Severity Scale
PSQI: Pittsburgh Sleep Quality Index
